# Venous thromboembolism prophylaxis guideline implementation is improved by nurse directed feedback and audit

**DOI:** 10.1186/1477-9560-9-7

**Published:** 2011-04-05

**Authors:** Harry Gibbs, John Fletcher, Peter Blombery, Renea Collins, David Wheatley

**Affiliations:** 1 Director of Cardiology, Lismore Base Hospital, Lismore, NSW, 2480 Australia; 2 Department of Surgery, Westmead Hospital, Westmead, NSW, 2145 Australia; 3 Honorary Cardiovascular Physician, Heart Centre, The Alfred Hospital, Melbourne, VIC, 3181 Australia; 4 Vascular Medicine Unit, Princess Alexandra Hospital, Brisbane, QLD, 4000 Australia; 5 Medical Affairs Clinical Operations, sanofi aventis australia pty ltd, Maquarie Park, NSW, 2113 Australia

## Abstract

**Background:**

Venous thromboembolism (VTE) is a major health and financial burden. VTE impacts health outcomes in surgical and non-surgical patients. VTE prophylaxis is underutilized, particularly amongst high risk medical patients. We conducted a multicentre clinical audit to determine the extent to which appropriate VTE prophylaxis in acutely ill hospitalized medical patients could be improved via implementation of a multifaceted nurse facilitated educational program.

**Methods:**

This multicentre clinical audit of 15 Australian hospitals was conducted in 2007-208. The program incorporated a baseline audit to determine the proportion of patients receiving appropriate VTE prophylaxis according to best practice recommendations issued by the Australian and New Zealand Working Party on the Management and Prevention of Venous Thromboembolism (ANZ-WP recommendations), followed by a 4-month education intervention program and a post intervention audit. The primary endpoint was to compare the proportion of patients being appropriately managed based on their risk profile between the two audits.

**Results:**

A total of 8774 patients (audit 1; 4399 and audit 2; 4375) were included in the study, most (82.2% audit 1; and 81.0% audit 2) were high risk based on ANZ-WP recommendations. At baseline 37.9% of high risk patients were receiving appropriate thromboprophylaxis. This increased to 54.1% in the post intervention audit (absolute improvement 16%; 95% confidence interval [CI] 11.7%, 20.5%). As a result of the nurse educator program, the likelihood of high risk patients being treated according to ANZ-WP recommendations increased significantly (OR 1.96; 1.62, 2.37).

**Conclusion:**

Utilization of VTE prophylaxis amongst hospitalized medical patients can be significantly improved by implementation of a multifaceted educational program coordinated by a dedicated nurse practitioner.

## Introduction

Venous thromboembolism (VTE), which comprises deep vein thrombosis (DVT) and pulmonary embolism (PE) represents a major public health problem. VTE is primarily a problem in hospitalized or recently hospitalized patients. The incidence of VTE has been shown to be more than 100 times greater among hospitalized patients than those in the community[1]. In Australia, VTE is estimated to complicate 2-3 per 1000 hospital admissions, but varies widely by principle diagnosis[2]. Moreover, postmortem studies indicate that approximately 10% of all hospital deaths are attributed to PE,[3-5] making it the most common preventable cause of hospital death[6].

In recent years, the prevention of VTE has been identified nationally and internationally as a priority area for improving patient safety. To support these efforts, a number of evidence-based guidelines have been made available which outline the appropriate use of prophylaxis to prevent VTE in a variety of patient populations [7-13]. Patients should be treated according to their individual risk and associated clinical conditions [7,11,14]. The Australian and New Zealand Working Party on the Management and Prevention of Venous Thromboembolism (ANZ-WP), which first convened in 1997, has sought to provide a practical pocket-sized booklet that summarised current best practice in VTE prevention. The most recently published version of these recommendations[15] is based on guidelines from the International Union of Angiology[8] and the American College of Chest Physicians (ACCP)[7] and state that every hospitalized adult patient should be assessed for their risk of VTE.

Despite the commonly held perception that VTE is a complication of major surgery, postmortem studies have shown that approximately 70% of fatal PEs occur in non-surgical patients[3-5]. Further, it is accepted in the literature that 50-70% of symptomatic thromboembolic events occur in non-surgical patients[7,14]. Nevertheless, the evidence base for clinical decision making regarding thromboprophylaxis in medical patients remains limited. Data from two meta-analyses demonstrate relative risk reductions of between 38% and 57% with the use of pharmacological prophylaxis depending on the endpoint being assessed[16,17]. These data have recently been corroborated by the National Health and Medical Research Council of Australia, who present relative risk reductions of between 39% and 60% with the use of pharmacological prophylaxis in general medical patients admitted to hospital[12].

Available published data from multinational observational studies demonstrate the underuse or suboptimal use of VTE prophylaxis to be a global problem. In the IMPROVE (International Medical Prevention Registry on Venous Thromboembolism) study, which assessed routine clinical practices in the provision of VTE prophylaxis in acutely ill hospitalized medical patients from 52 hospitals in 12 countries, only 60% of patients who met the criteria for prophylaxis actually received it [18]. The global ENDORSE (Epidemiologic International Day for the Evaluation of Patients at Risk for Venous Thromboembolism in the Acute Hospital Care Setting) study evaluated over 68,000 patients in 365 hospitals across 32 countries, again showing that amongst the surgical patients at risk only 58.5% received ACCP-recommended VTE prophylaxis whilst this was even lower (39.5%) amongst at-risk medical patients [19]. Further analysis of the ENDORSE study data has shown that whilst the use of prophylaxis differs between countries, in general its use appears to be associated with disease severity rather than medical diagnosis [20].

This global picture also extends to Australia. A study of 250 surgical patients at the Royal Hobart Hospital, Tasmania, found that only 59.2% of patients had received appropriate prophylaxis according to the hospital's approved guidelines [21]. Amongst patients at high risk of VTE, only 25.7% were prescribed the recommended preventive measures. Similarly, in 2002 it was reported that the majority of inpatients in The Canberra Hospital (TCH) were not receiving appropriate prophylaxis according to international guidelines [22,23]. More recently, in the ENDORSE study, 80% of surgical patients were at risk for VTE yet only 72% received ACCP-recommended treatment. Consistent with global trends medical patients again fared worse with only 42% of those at risk receiving ACCP-recommended treatment [19].

There is a clear need for improved implementation of existing guidelines. Various strategies have been employed and their effectiveness systematically reviewed [24,25]. Passive dissemination of guidelines was found to be the least effective method whilst the most effective strategies combined a system of active education of health providers, the use of reminders to assess for VTE risk and iterative audit and feedback to enable a continuous cycle of quality improvement. When such a process was employed at the Royal Hobart Hospital, Tasmania, a significant increase in adherence to guidelines resulted, the biggest improvement being amongst patients at high-risk (from 25.7% pre-intervention to 76.5% post-intervention) [21]. Similarly, data collected in TCH over the period 2001-2005 as part of a quality improvement program highlighted that, at baseline, there was a clear absence of policies to assess and respond to patient risk. This improved over the duration of the study; there was a statistically significant increase in the use of risk assessment in the ward setting (from 7.7% to 100%) and in the extent of coverage of patients with anticoagulant prophylaxis (from 48% to 74%)[23].

In this paper we report the results of a multicentre clinical audit study examining the effect of a dedicated VTE nurse educator on the use of prophylactic measures in acutely ill medical patients at 15 hospitals across Australia. The specific aims of the audit were to determine the extent to which appropriate VTE prophylaxis is being utilized at baseline and to examine the impact of a multifaceted program on the rate of appropriate VTE prophylaxis.

## Methods

The VTE Task Force Audit was a multicentre clinical program performed in 15 hospitals throughout Australia, and conduced over the period June 2007 to August 2008. The use of VTE prophylaxis in acutely ill medical patients was assessed prior to and following an interventional program implemented by a dedicated VTE nurse educator. The audit was conducted in accordance with the Declaration of Helsinki as amended in 2004 [26] and written approval was obtained from the relevant human research ethics committee at each study site. Each study site performed a baseline audit of the VTE risk and administered prophylaxis on 200-300 consecutively presenting adult (≥18 years of age) patients admitted to hospital for an acute medical illness with an in-hospital stay of 3 days or more. A full time nurse educator then instituted a multi-faceted program of system change comprising active educational sessions, paper and verbal reminders and feedback of the initial audit results. A second audit of another 200-300 consecutive medical patients was performed four months after the institution of the program. The proportion of patients receiving appropriate VTE prophylaxis was compared at baseline and following the program to assess its efficacy. Full details of the study methodology (patient selection, data collection, primary endpoints, educational program, analyses and statistical analysis) have been published previously in a design paper [27].

**Figure 1:**
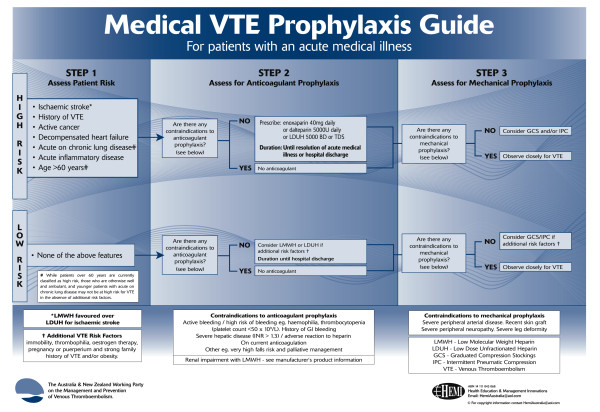
**VTE risk classification in medical patients**. Reproduced with permission from: The Australia and New Zealand Working Party on the Management and Prevention of Venous Thromboembolism. Prevention of venous thromboembolism: best practice guidelines for Australia and New Zealand. 4th Edition. 2007. Sydney, Health Education and Management Innovations.

Recommendations for the prevention of VTE issued by the ANZ-WP [15] (ANZ-WP recommendations) were used for assessment of risk and suitability for anticoagulant or mechanical prophylaxis. For the purposes of our audit, medical patients were classified as being at either "high" or "low" risk, as defined at step 1 in the ANZ-WP recommendations (Figure 1)[15]. All analyses are based on the intent to treat population, which comprised all patients for whom a case record form was submitted.

Results

A total of 8774 patients (audit 1; 4399 and audit 2; 4375) from 20 clinical units within 15 hospitals were included in the study. Each hospital met the target recruitment criteria of 200-300 patients for each audit (Table 1). The demographic profiles of patients included in the baseline and post intervention audits were similar (Table 2).

**Table 1:** Contribution of patients from individual study sites.

Hospital	Baseline (Audit 1)	Post intervention (Audit 2)
Ballarat Hospital	236 (5.4)	300 (6.9)
Concord Repatriation General Hospital	300 (6.8)	299 (6.8)
Dandenong Hospital	300 (6.8)	300 (6.9)
Fremantle Hospital	300 (6.8)	267 (6.1)
Greenslopes Hospital	298 (6.8)	297 (6.8)
Liverpool Hospital	297 (6.8)	300 (6.9)
Mater Adult Hospital	299 (6.8)	239 (5.5)
Monash Medical Centre	300 (6.8)	300 (6.9)
Nambour Hospital	301 (6.8)	297 (6.8)
Queen Elizabeth Hospital	270 (6.1)	278 (6.4)
Royal Brisbane Hospital	298 (6.8)	298 (6.8)
Royal North Shore Hospital	300 (6.8)	300 (6.9)
St George Hospital	300 (6.8)	300 (6.9)
Western Hospital	300 (6.8)	300 (6.9)
Westmead Hospital	300 (6.8)	300 (6.9)
TOTAL	4399 (100)	4375 (100)

All values presented as number (%).

**Table 2:** Patient demographics.

	Baseline (Audit 1)	Post intervention (Audit 2)
Gender		
Male, n (%)	2243 (51.0)	2211 (50.5)
Female, n (%)	2156 (49.0)	2164 (49.5)
Mean age in years (SD)	69.0 (18.26)	69.7 (17.68)
Mean weight in kg (SD)	74.6 (21.9)	74.3 (23.3)
High risk*, n (%)	3618 (82.2)	3544 (81.0)

n = number; SD = standard deviation *High risk = Age >60 years or admission due to acute respiratory disease, heart failure, malignancy, ischaemic stroke, rheumatological or inflammatory or existence of previous VTE[15].

**Figure 2:**
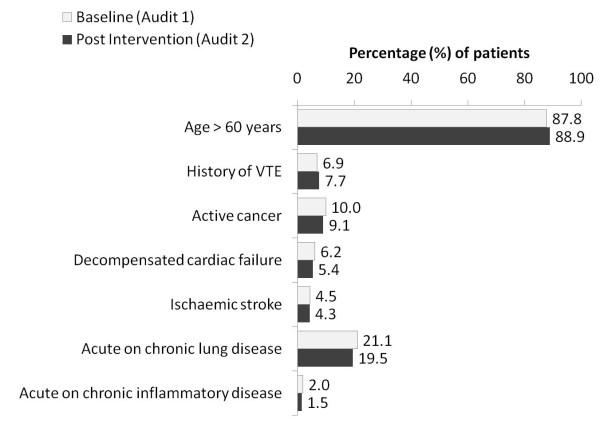
**Risk factors for VTE based on ANZ-WP best practice recommendations**[15].

The majority of patients (audit 1; 3618 [82.2%] and audit 2; 3544 [81.0%]) were classified as high VTE risk based on the ANZ-WP recommendations. Amongst those patients classified as being at "high-risk", the most frequent of the risk factors were age >60 years (87.8% and 88.9% for audits 1 and 2, respectively), acute or chronic lung disease (21.1% and 19.5% for audits 1 and 2, respectively) followed by active cancer (Figure 2). The presence of multiple risk factors amongst these patients was common and similar in each audit; 63% and 66% in audits 1 and 2, respectively had one risk factor, 35% and 32%, respectively had two risk factors and 2% and 2%, respectively had three risk factors.

Prophylaxis protocols were in place in 44.2% (69/156) of the hospital units involved in audit 1 and 49.3% (67/136) of those involved in audit 2. Amongst hospital units that did not have a prophylaxis protocol in place at audit 1, 5.3% had introduced a protocol by the commencement of audit 2 (95% CI 2.1% to 10.5%, P < 0.0001). One third (5/15) of hospitals had prophylaxis protocols in all of their units for both audits. Protocols were in place for both audits in all of the gastroenterology, renal medicine, rheumatology, geriatric and stroke units of all participating hospitals.

Contra-indications to anticoagulant prophylaxis were common (21.4% and 26% in audits 1 and 2, respectively). The most common contraindication in both audits was current therapeutic LMWH/UFH/warfarin; which accounted for almost half of all contraindications (audit 1: 10.5% and audit 2: 11.7%). Contra-indications to anticoagulant prophylaxis were more common in patients classified as being at high VTE risk than in those at lower risk.

**Figure 3:**
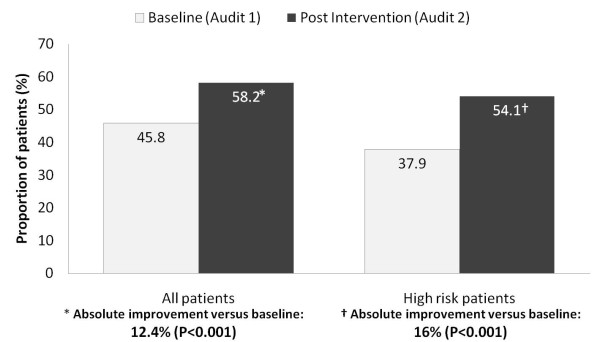
**Proportion of patients receiving appropriate VTE prophylaxis in the baseline and post intervention audits**.

The primary endpoint of this study was to determine whether or not a patient was being appropriately managed based on their VTE risk profile. The proportion of patients being treated according to the ANZ-WP recommendations increased significantly between audits (Figure 3); the absolute improvement versus baseline was 12.4% (95%CI 8.8-16.1, p < 0.001) in all patients and 16.1% (95%CI 11.7-20.5, p < 0.001) in patients classified as being at high risk. The improvement in appropriate prophylaxis in high risk patients occurred due to an increase in both the number of patients prescribed anticoagulants and also in the number receiving compression stockings [audit 1 7.6% versus audit 2 18.1%; absolute change 10.5%; 95%CI 5.8-15.2] in whom a contra-indication to anticoagulants existed. In audit 1, a higher percentage of patients were being treated with enoxaparin (16%, 95%CI 12.8-19.3) than unfractionated heparin (10.9%, 95%CI 8.0-13.8), whereas in audit 2, enoxaparin (17.3%, 95%CI 12.2-22.4) and unfractionated heparin (17.3%, 95%CI 11.8-22.7) were used equally.

The likelihood of any patient being treated according to ANZ-WP recommendations increased significantly as a result of the nurse educator program; odds ratio (OR, 95% CI) 1.68 (1.45, 1.94). Amongst patients classified as high risk, the odds of being treated according to the recommendations as a result of the nurse education program was nearly 2 (OR 1.96; 1.62, 2.37) and was higher when a unit protocol was present (OR 2.46; 1.85, 3.28).

## Discussion

Our study evaluated the use of thromboprophylaxis amongst almost 9000 acutely ill medical patients admitted to 15 hospitals across Australia. At baseline fewer than 2 out of 5 patients classified as being at high risk were receiving appropriate VTE prophylaxis. Feedback of the baseline results to the relevant medical, nursing and allied health staff and education implemented by a dedicated VTE nurse educator at each hospital resulted in significant improvements in patient care. As a result of this multifaceted education program, the likelihood of a high risk patient being managed appropriately increased by 42%.

Clinical audits, whilst valuable educational tools, are not without their limitations. Our study utilised a simple before and after design, which may be subject to methodological limitations[28]. Confounding issues, such as the relative experience of the medical staff at the time of each audit were likely to be minimal given that the first cycle of data collection occurred mid-year when trainees would have had at least 6 months experience. The proportion of patients audited in each cycle did not differ significantly either by hospital unit or reason for admission. Having only one post-implementation data point makes it difficult to determine whether the improvements we observed would be maintained or improved upon over the longer term. Ongoing support appears to be a a key component of success in this respect. Gallagher et al reported an initial increase in VTE risk assessment rates, followed by a fall and then a further increase (to 100%) over a 4-year period of ongoing data collection, feedback and education[23].

VTE can lead to serious illness or death, long term morbidity, prolonged length of stay, and increased hospital costs. The goals of VTE prophylaxis - risk assessment followed by prescription of appropriate prophylaxis - are well established. It is further established that multiple active strategies which incorporate reminders for clinicians to assess patients for VTE risk and assist in the selection of appropriate prophylactic measures are more likely to result in improvements[24,25]. However, to effect change in VTE prophylaxis practice requires a combination clinical leadership, improved clinician knowledge of risk assessment and appropriate prescribing, and a supportive system which embeds VTE prophylaxis into routine care processes.

In the US, it has been mandated that hospitals use formalised tools for VTE risk assessment and provide appropriate prevention measures[29,30]. Guidelines, protocols and VTE prevention programs have been implemented in Australia, but to date mandatory order sets or risk assessment protocols have not been established and clinician preference appears to be a key driver in the prescription of prophylaxis.

It has been suggested that through leadership and education nurses are ideally placed to play a central role in the implementation of changes to ensure that patients are assessed and the most appropriate thromboprophylaxis selected, prescribed and delivered[31]. Our study is the first multicentre clinical audit conducted to determine the extent to which VTE risk is assessed and managed in acutely ill medical patients in Australia. We have demonstrated that, via the employment of a full-time prophylaxis nurse, a combination of audit and feedback, education and visual reminders significantly increased the rate of appropriate VTE prophylaxis in acutely ill hospitalized medical patients. The results, whilst not providing all the answers to this important clinical issue, provide encouragement and support to those seeking to implement change. Cost-efficacy data will be required to determine the net long-term benefits of employing a full-time prophylaxis nurse and would also likely be required before mandatory systems are established.

## Conclusion

Our results confirm prior findings that, despite the availability of management guidelines and prophylaxis protocols, VTE prophylaxis is underutilised in acutely ill hospitalised medical patients in Australia. Implementation of a multifaceted educational program coordinated by a dedicated nurse practitioner resulted in significant improvements in appropriate prophylaxis. Amongst acutely ill medical patients at high risk of VTE, the likelihood of being treated according to guideline increased significantly as a result of this nurse education program.

## Competing interests

This work has been carried out with financial support from sanofi-aventis Australia pty ltd, who markets the low molecular weight heparin product enoxaparin sodium under the brand names Clexane^® ^and Clexane Forte^®^. David Wheatley is an employee of sanofi-aventis australia pty ltd.

## Authors' contributions

HG: Study design, acquisition of data, analysis and interpretation of data, drafting of the manuscript and critical revision of the manuscript.

JF: Study design, acquisition of data, interpretation of data and critical revision of the manuscript.

PB: Study design, acquisition of data, interpretation of data and critical revision of the manuscript.

RC: Study design, acquisition of data, interpretation of data and critical revision of the manuscript.

DW: Study design, acquisition of data, analysis and interpretation of data and critical revision of the manuscript.

All authors read and approved the final manuscript.
